# Therapies from Fucoidan; Multifunctional Marine Polymers

**DOI:** 10.3390/md9101731

**Published:** 2011-09-30

**Authors:** Janet Helen Fitton

**Affiliations:** Marinova Pty Ltd., 249 Kennedy Drive, Cambridge, Tasmania 7170, Australia; E-Mail: Helen.fitton@marinova.com.au; Tel.: +61-3-62485800; Fax: +61-3-62484062

**Keywords:** fucoidan, inflammation, fibrosis, viral infection

## Abstract

Published research on fucoidans increased three fold between 2000 and 2010. These algal derived marine carbohydrate polymers present numerous valuable bioactivities. This review discusses the role for fucoidan in the control of acute and chronic inflammation via selectin blockade, enzyme inhibition and inhibiting the complement cascade. The recent data on toxicology and uptake of fucoidan is detailed together with a discussion on the comparative activities of fractions of fucoidan from different sources. Recent *in vivo*, *in vitro* and clinical research related to diverse clinical needs is discussed. Targets include osteoarthritis, kidney and liver disease, neglected infectious diseases, hemopoietic stem cell modulation, protection from radiation damage and treatments for snake envenomation. In recent years, the production of well characterized reproducible fucoidan fractions on a commercial scale has become possible making therapies from fucoidan a realizable goal.

## 1. Introduction

Fucoidans are a class of sulfated, fucose-rich polymers found in brown macroalgae [1,2]. They were identified in the first half of the last century by Kylin [3]. Subsequent research by researchers such as Bird [4] and Pervical [5] confirmed the basic identity of this class of compounds. Black reports the systematic isolation of fucoidan from a number of different British seaweeds [6]. Fucoidan was recognized as having a role in the biology of the seaweed, and examined over the next few decades for its activity in a number of biological systems. Current research interest in fucoidan is global. Research occurs in Australia, Japan, Korea, Russia and China in addition to Europe and the Americas. The intensity of biological activities of fucoidan varies with species, molecular weight, composition, structure and the route of administration.

Despite notable bioactivities and a lack of oral toxicity, fucoidans remain relatively unexploited as a source of therapeutics due to their plant source and heterogeneity. In recent decades pharmaceutical companies have preferred “druggable” well defined smaller molecules, leading to a neglect of the opportunities for larger and polydisperse entities. Older naturally occurring polymeric drugs such as heparin have held their place in the modern pharmacopeia despite recent concerns [7]. Carefully controlled sources of seaweed together with modern extraction and characterization methods to create reproducibly defined products together with new regulatory avenues mean that fucoidan fractions can be produced to standards suitable as ingredients in medical devices and even cross the threshold into drug development route [8]. There are also further potential applications for fucoidan products in complementary medicine and topical applications.

The reading of the literature can be confusing due to the diverse sources and fractions of fucoidan used. In general, fucoidans contain a high proportion of fucose in the sugar backbone of the polymer. They are sulfated, may be acetylated and may also contain uronic acids. The yield of a crude first fraction of fucoidan is generally 2–10% by weight, although *Cladosiphon* species may yield over 20% by dry weight. Fucoidans derived from seaweeds are all highly branched. A second source of linear rather than branched fucoidans is echinoderms, in particular sea cucumbers, which are largely outside of the scope of this review. Earlier reviews on the structure and significance of the fucoidans can be found in Fitton [9], Pomin [10] and Berteau [2] and also by Li [11].

The most common source of experimental fucoidan used in the literature is derived from *Fucus vesiculosis*, a common harvest crop of a northern hemisphere kelp. This fucoidan, like all primary fucoidan extracts, is heterodisperse and well characterized in an elegant paper by Nishino [12]. *Fucus vesiculosis* fucoidan is a high fucose sulfated fucoidan. Other common fucoidans are sourced from edible species such as *Cladosiphon okamuranus*, *Laminaria japonica* and *Undaria pinnatifida*. These seaweeds are harvested in significant quantities across Asia and have an excellent toxicity profile. The backbone sugars are different again. *Undaria pinnatifida* fucoidan contains a high proportion of galactose for example.

This review begins by identifying new research in uptake and toxicity of fucoidans. Secondly, this review limits the scope of potential therapeutic use of fucoidan in inflammation related areas; injury, infection, chronic inflammation and fibrosis, and lastly, protection of neuronal function. These areas represent areas of need for new approaches and perhaps greater potential for commercialization of new technology. It is not inferred that other areas of fucoidan bioactivity are not important, or indeed, any less likely to lead to real world therapeutic use. Research into the cancer inhibitory effects of fucoidan fractions is reviewed elsewhere [2,9] and not within the scope of this review.

## 2. Uptake and Fate of Fucoidan

The yet unanswered question is “Where does fucoidan go after oral, intraperitoneal or intravenous administration?” Although a number of research papers indicate biological effects after oral ingestion or systemic delivery, very little research has taken place on the uptake and fate of fucoidan. The expectation that large molecules are not orally absorbed causes difficulties in understanding how apparently systemic effects occur. Early work on digestion of fucoidan suggested that it was not changed by human bacterial flora and was wholly excreted [13,14]. Perhaps systemic observations are partly a result of prebiotic effects? Recent research indicates that there are favorable changes in intestinal flora after ingestion of fucoidan, including increased Lactobacilli [15,16].

There is however, a growing body of evidence for the absorption of larger polysaccharide molecules such as chondroitin sulfate [17] and polyethylene glycol [18], albeit in small quantities. The more recent research by Irhimeh [19], Tokita [20] and Nakazato [21] strengthens the case for small amounts of systemic uptake from oral dosing in which the fucoidan is unchanged in the serum.

Irhimeh used an antibody based method to detect small amounts of orally ingested Undaria derived fucoidan in serum in a human clinical study [19]. More recently, Tokita indicated that orally ingested *Cladosiphon* derived fucoidan can be detected in serum in a rat model [20] at levels two orders of magnitude lower than observed by Irhimeh. The differences in observations on the serum levels may be accounted for by differences in the source of fucoidan used, the time span over which the observations were taken (hours as opposed to days of ingestion) and the detection method (specific rather than cross reacting antibodies). Tokita also recorded molecular weight profiles of the fucoidan when absorbed, showing that fucoidan molecular weight was unchanged in serum or plasma. Lower molecular weight fucoidan was observed in the urine, which may indicate a hitherto unknown mammalian mechanism for the breakdown of fucoidan. The accumulation of sulfated polysaccharides in the kidney, and their urinary excretion was noted by Guimaraes in 1997 [22] although in this case, the fucoidan was delivered intravenously.

Nakazato [21] examined the fate of a crude (average 28 kDa) and a higher molecular weight subfraction (41.4 kDa) of *Cladosiphon okamuranus* derived fucoidan administered in the drinking water at 2% w/v in a rat model, determining the concentration of fucoidan in the liver. This study clearly demonstrated an inhibitory effect of orally delivered higher molecular weight fucoidan on induced fibrosis of the liver which was associated with decreased tissue expression of transforming growth factor beta (TGF beta) and stromal derived factor (SDF1 also known as CXCL12), the receptor for CXCR4. This research was important, not only because it illustrated the fibrosis inhibitory potential of fucoidan, but demonstrated systemic distribution of the molecule after oral ingestion in this animal model.

All of these studies have used antibody technology to either stain or quantify fucoidan. The increasing availability of antibodies will facilitate this type of research. Mizuno also prepared monoclonal antibodies that reacted with fucoidan isolated from *Laminaria japonica* and *Kjellmaniella gyrate*, but not with fucoidan from *Undaria pinnatifida* [23]. However, these were used only to stain for fucoidan in seaweed preparations. Earlier researchers also developed antibodies to *Fucus vesiculosis* fucoidan for botanical purposes [24]. The data are summarized in Table 1.

## 3. Toxicology

Fucoidan derived from all species appears to lack toxicity *in vitro* and *in vivo*. In recent toxicology reports, fucoidan derived from *Undaria pinnatifida* and *Laminaria japonica* was found to be safe in animal models at very high levels of ingestion [25,26]. The oral safety of fucoidan is echoed in clinical studies. Human clinical studies using orally ingested fucoidan have shown no toxicity at a dose of 1 g per day up to three months [27], or 3 g per day for 12 days [28,19]. It is interesting- and important-to note that fucoidan has substantial coagulation modifying activity *in vitro*. This activity was echoed in Irhimeh 2009 [29] in which significant decreases in global clotting time were noted after ingestion of 3 g *Undaria pinnatifida* fucoidan although these stayed within the normal clinical range. Clotting times were increased at higher doses of 900 mg/kg body weight in a rat model with *Laminaria japonica* fucoidan [26], but intriguingly, no change in clotting time was noted in a rat model in which *Undaria pinnatifida* fucoidan at 1350 mg/kg body weight was ingested [30]. See Table 2 for details.

## 4. Differences between Fucoidan Fractions

One difficulty in making comparisons within the literature lies in the many variations to molecular weight and composition of the fucoidan fractions used. Fractions vary widely in terms of purity, composition, weight and sulfation even from the same species of macroalga. As polyphenols and other components present in macroalgae also have bioactivity, less pure fractions can deliver confounding data [9,35].

A recent multi-author paper compared fucoidans from different sources in a suite of bioassays [36]. Cumashi and colleagues comprehensive study uses comparable preparation techniques to generate samples and concentrated on commercial (or potentially commercial) sustainable sources of seaweeds. Their data thoroughly illustrates the common bioactivities of fucoidans and yet their differences. All fucoidans tested inhibited *leukocyte* recruitment in an inflammation model in rats. The model used consisted of an induced acute peritonitis in female Wistar rats and the measurement of neutrophils into the peritoneal cavity. Test fucoidans were delivered intravenously. Fucoidans are well known as L-selectin blockage agents and were found to inhibit neutrophil extravasion into the peritoneal cavity. Clotting parameters were also assessed. The most active inhibitors were fucoidans from *Laminaria saccharina* and *Fucus evanescens*, which inhibited neutrophil extravasation by more than 90% whereas fucoidan from *Fucus distichus* and *Fucus spiralis* inhibited the neutrophil transmigration by only 60%. Other parameters investigated included P selectin inhibition in a platelet aggregation model, coagulation parameters and cancer cell adhesion to platelets *in vitro*. All fucoidans, except that from *Cladosiphon okamuranus*, exhibited anticoagulant activity *in vitro* and *in vivo*. Fucoidans from *Laminaria saccharina, Laminaria digitata*, *Fucus serratus*, *Fucus distichus*, and *Fucus vesiculosus* effectively inhibited breast carcinoma cell adhesion to platelets, an effect which may point to an inhibition of “platelet cloaking”, a mechanism used by cancer cells to metastasise unchallenged throughout the body [37].

Others have investigated the relative effects of higher and lower molecular weight fractions of the same fucoidan source and found significant differences in their bioactivity. Firstly, Nakazato found that an orally delivered higher molecular weight fraction of fucoidan was more effective than an unfractionated crude fucoidan in inhibiting liver fibrosis [21]. Secondly, Maruyama confirmed that fucoidan is effective as an anti-tumor and immune modulating agent [38,39]. Shimizu then orally administered three different preparations of fucoidan from *Cladosiphon okamuranus* to mice [40]. The key findings were increases in CD8 expression in spleens of the highest molecular weight fucoidan fed mice. The CD4/CD8 ratio tended to decrease, and the number of cells expressing CD11b cells (NK, monocytes and macrophage cells) tended to increase as compared to the lower molecular weight fucoidan fed mice.

Thirdly, Park examined the differential effects of high and low molecular weight fractions of fucoidan in an arthritis model [41]. There was a marked contrast in the two preparations. The high molecular weight fraction activated the inflammatory process, whereas the low molecular weight fraction inhibited the disease.

Snake envenomation may be ameliorated by fucoidan (see Section 6.15. In a experiment to determine whether lower molecular weight would increase bioavailability of fucoidan and its effectiveness, Azofeifa found the reverse [42]. High molecular weight fucoidan was necessary to achieve the therapeutic effect against crude venom *in vivo*, whereas the lower molecular weight preparation was ineffective, despite being equally effective *in vitro*.

Lastly, the way in which fucoidan is administered may make a difference to activity. This is well illustrated in research presented by Yanase who describes the suppression of an IgE response to albumin by intraperitoneal fucoidan [43]. Elevated serum levels of IgE towards common environmental allergens are a feature of allergic diseases such as asthma and atopic dermatitis. Having found that fucoidan inhibited IgE production by B cells *in vitro*, they studied its effect in an allergic model in mice. In this study, the stimulated increase of plasma IgE was suppressed when fucoidan was delivered intraperitoneally at 100 mcg per mouse, but not when delivered orally at 10 mg per mouse. It is interesting to compare this to Hayashi’s study in which orally delivered fucoidan was effective in reducing viral titres in mice after influenza infection [44].

These recent examples are illustrative of the variation in fucoidan and the care that needs to be taken when developing a fraction for a particular application. They also illustrate remarkable bioactivity by molecules with low toxicity profiles.

## 5. Inflammation and Fibrosis

Inflammation can be classified as either acute or chronic. Acute inflammation, which may occur as a result of infection or injury, is characterized by its rapid resolution, neutrophilic inflammation and edema. Chronic inflammation is a response that persists for several months and is characterized by simultaneous inflammation, tissue remodeling and repair processes without resolution. Whilst there are clinically distinct fibrotic disorders they commonly start off from a persistent aggravating inflammation that sustains the production of matrix degrading enzymes, growth factors and cytokines, which gradually obliterate normal tissue architecture and cause organ damage [45].

Acute inflammatory responses experienced after an ischemic event such as a heart attack, can be destructive to organs. Post ischemic organ damage results from inflammatory cascades pursuant to necrotic tissue. Diseases involving chronic inflammation are debilitating. Causes of inflammation are diverse, ranging from viral infection to systemic metabolic disorders. Chronic inflammation can enhance cancer metastasis and lead to increases in other disease processes. Non toxic, orally delivered therapeutics to address both short term and long term inflammation are highly desirable.

Fucoidan’s potential lies in its pleiotropic anti-inflammatory effects. These include inhibition of selectins, inhibition of complement and enzyme inhibitory activity.

### 5.1. Inhibition of Selectins

Fucoidan is a well known research tool as a selectin blockade agent [36,46–54]. Selectins are cell surface receptors on white blood cells that perform a braking function for the cells, allowing them to roll on an organs endothelial surface and ultimately enter that tissue space. Fucoidan prevents P and L selectins and thus *leukocyte* adhesion and rolling thereby reducing inflammation in whole organs such as the heart, kidneys or liver. The relative selectin blocking effects of fucoidans from different sources was investigated by Cumashi and colleagues [36]. These are discussed earlier in this review. All fucoidans tested were effective at inhibiting neutrophil extravasion in a peritoneal inflammation model system.

The selectin blockade effect has been established *in vivo* as a potential therapy for the prevention of post ischemic reperfusion injury and autoimmune cardiac injury [51]. In Tanaka’s surgical model, saline or fucoidan was delivered intraperitoneally to rats over 28 days with either a sham operation or with cardiac myosin injection to stimulate autoimmune myocarditis. The invasion of CD4-positive T cells into the myocardium is the initial pathology in this model. After 3 weeks, fucoidan treatment improved the function of the heart muscle and its weight when compared with the control. Fucoidan treatment decreased serum inflammatory markers, the myocarditis area and inhibited macrophage and CD4-positive T-cell infiltration into the myocardium.

In 2010, Thomes investigated the effects of *Cladosiphon okamuranus* fucoidan in a model of cardiac infarction (*i.e.*, a heart attack) induced by isoproterenol [52]. Oral dosing of *Cladosiphon* derived fucoidan at 150 mg/kg for 7 days had a marked protective effect on the sequelae of the infarction, including lowering cardiac enzymes and lipid profiles. Like many other researchers, Thomes debates whether the effects are via absorption or perhaps conferred via immune changes in the gut.

In well-designed research by Kubes and colleagues, the mechanism of post ischemic organ protection by *Fucus vesiculosis* fucoidan was shown not to be a simplistic one [47]. Kubes used intravital microscopy to look at reperfusion of the liver after an ischemic event or endotoxin exposure. Fucoidan effectively inhibited neutrophil entry after ischemia but not after endotoxin exposure. Kubes noted that liver ischemia also leads upstream to gut ischemia and hypothesized that the protection of the liver was actually a result of protection of the gut, preventing downstream inflammation of the liver. After rerouting the circulation and removing the intestines in the rat model, fucoidan did not protect against neutrophil adhesion in the liver to the same degree, confirming that the protective effect is partly due to inhibition of inflammation in the gut.

Most recently, experimental encephalomyelitis was reduced by administration of fucoidan [53]. Kim used 50 mg/kg, daily intraperitoneal administration of fucoidan into a rat to test whether it could either treat or prevent encephalomyelitis generated by immunization with guinea-pig myelin basic protein. Preventive dosing was carried out one day before immunization and the 6 days thereafter. Treatment dosing was carried out from the 8th to the 14th day of the disease. Surprisingly, prevention was not successful in contrast to treatment, which was highly effective. The lessening of clinical effects coincided with decreased infiltration of inflammatory cells in the affected spinal cord, probably partly by L selectin blockade and the suppression of specific CD4+ T cells.

P selectin is present on platelets and assists with aggregation. Xenografting (the transplant of an organ from one species to another) is often complicated by abnormal clotting responses. In an attempt to overcome this, Alwayn [54] investigated the effects of a number of agents on xenograft induced clotting in a baboon model. Fucoidan from *Fucus vesiculosis* was very successful in inhibiting the platelet aggregation although the perturbation of clotting was considered problematic. Alwayn did not consider the potential to inhibit complement activation, although this is known limiting factor in xenografts [55].

### 5.2. Inhibition of Complement

Complement is a cascade system of enzymatic proteins occurring in normal serum that help to regulate immunity and form part of the pathogen defense system [55]. It consists of “classical” and “alternative” pathways that are triggered by, and combine with antibody-antigen complexes, producing lysis in affected cells. Complement consists of approximately 30 proteins, known as C1 to C9, with subtypes for each protein. Components C3 and C5 are involved promotion of leukocyte chemotaxis whereas C1 and C4 are involved in the specific antibody response to viruses. The alternative complement pathway is sequence in which C3 and C5 to C9 are activated without participation by C1, C2 and C4 or the presence of an antibody-antigen complex. Fucoidan fractions are very effective inhibitors of this cascade [55–59] being at least 40 times more potent than heparin.

Fucoidan can block the formation of classical C3 convertase. This may prevent the production of the pro-inflammatory anaphylatoxins and further downstream in the cascade, the C3b fragment. Indeed, this is one of the possible mechanisms via which fucoidan exerts an anti-allergic effect, as observed in diverse experimental models.

Tissot demonstrated that fucoidan strongly binds to C1q and points out that inhibition of complement binding to C1q receptors present on endothelial cells could assist with xenograft (cross species transplants) rejection [56,57]. Indeed, fucoidan derived from *Ascophyllum nodosum* protected of porcine endothelial cells against the complement-mediated lysis that is responsible for rejection [56]. More recent research on xenograft protection found that *Fucus vesiculosis* fucoidan was useful inhibitor of platelet aggregation—as a result of selectin blockade, although concerns were raised over its temporary anticoagulant effect. Curiously, complement blockade was not considered in this research [54].

Fucoidans are known to be highly branched polysaccharides but the potential meaning of the branching structures in terms of bioactivity was not considered until Clement’s research of 2010 [60]. In an elegant NMR study, Clement tackles the structural heterogeneity and the random sulfation of the polysaccharide chain with relation to how they affect complement binding and inhibition. The results suggest that branching of fucoidan oligosaccharides is a major factor in their anti-complementary activity at the conformational level. This work is significant as it demonstrates that not only molecular weight, sulfation and sugar composition affect bioactivity, but also three dimensional structure. When therapies from fucoidan are developed, a holistic approach to all of these parameters is essential.

### 5.3. Inhibition of Enzymes

Fucoidan also has significant enzyme inhibitory activity against a number of enzymes including matrix metalloproteases, hyaluronidases and elastases [61,62]. This inhibitory activity limits tissue breakdown in inflammatory settings caused by injury and disease and can even inhibit metastasis.

Ultraviolet radiation is a prominent cause of skin photoaging and even skin cancers. It causes marked increases in elastase activity, breaking down elastin fibers, which accelerates loss of skin condition [63]. Research reported in 2009 indicates that ultraviolet radiation injury to skin fibroblasts *in vitro*, which causes increase in matrix metalloproteases and reduces procollagen levels, is markedly reduced by the presence of *Fucus vesiculosis* fucoidan [64]. Senni reported a comprehensive study on the inhibitory effects of fucoidan. Using human skin in *ex vivo* experiments he showed that fucoidan was able to curtail human leukocyte elastase activity which protected the elastic fibers from degradation [62]. A clinical study also indicated good potential for amelioration of skin aging [65]. *Fucus vesiculosis* fucoidan was applied to skin at 1% w/v twice daily for five weeks. A significant decrease in skin thickness and improvement in elasticity was noted compared to controls.

According to new research by Hlawaty [66], matrix metalloprotease type 2 (MMP2) is responsible for intimal hyperplasia in a rat model. In this model, the aorta is damaged and the rats are treated via intramuscular injection with 5 mg/kg/day of a low molecular weight fucoidan derived from *Fucus vesiculosis* for two weeks. The proliferation of the smooth muscle walls which causes the narrowing of the lumen in the damaged vessel was measured and found to be considerably more open in the fucoidan treated rats. Previous research has shown that this fucoidan fraction can successfully inhibit smooth muscle proliferation [67]. This study then looked at the enzyme activity in the vessel, and found that fucoidan specifically inhibited MMP2, an enzyme which degrades type IV collagen, a major structural component of basement membranes.

## 6. Therapies from Fucoidan

Selected applications for fucoidan based therapies are discussed in this section. Clinical data together with promising *in vivo* and *in vitro* data is discussed with reference to a diverse set of therapeutic goals.

### 6.1. Osteoarthritis

Osteoarthritis is the most common and disabling form of arthritis experienced by an increasing number of people as the population ages. It is characterized by a slow and progressive degeneration of articular cartilage. Whilst it is often regarded as a disease of “wear and tear” through ageing, osteoarthritis has a strong genetic component and is also affected by injury and by diseases such as obesity. In addition to the cartilage itself, osteoarthritis also involves the surrounding tissues, including the synovium and the subchondral bone. Osteoarthritis has an inflammatory component, and undoubtedly, a powerful tissue breakdown component which result in pain and stiffness [68].

Several recent studies indicate a role of fucoidan in addressing the symptoms of osteoarthritis. Park *et al.* used an animal model of collagen induced arthritis, and showed that orally administered *Undaria pinnatifida* fucoidan successfully inhibited pain [41]. In a small human clinical study, osteoarthritis symptoms were significantly inhibited by oral administration of fucoidan rich seaweed extract. Over three months, symptoms were reduced by 52% [27]. This result is a marked improvement for osteoarthritis symptoms. There was no reduction in TNF alpha which was used as inflammation marker, but an accompanying study in healthy volunteers showed a decrease in Interleukin 6, a marker for chronic inflammation [33].

The mechanism for the clinically observed reduction in pain of osteoarthritis is unclear. Was pain decreased by the blockade of neutrophils? Cunha showed that fucoidan successfully blocked the accumulation of neutrophils and pain nociception in an animal model [69]. Cunha’s study emphasized the importance of neutrophils as an originator of inflammatory hypernociception and confirmed that fucoidan blocks the accumulation of neutrophils by selectin blockade in their model. See Table 3.

### 6.2. Surgical Adhesions

As identified earlier, fucoidan is a potent selectin blocker, and can therefore inhibit inflammatory damage, such as that caused by post-ischemic inflammation. A fibrotic reaction after the inflammation is responsible for a condition called “surgical adhesions”. This condition is a disabling result of minor damage during abdominal surgery or sometimes after infections. Small injuries cause inflammatory responses on the external surfaces of the bowel which generate the fibrotic response, leading to areas in which the bowel becomes tied to the peritoneal wall or to other parts of the bowel. Products aimed at reducing post surgical adhesions have variable levels of success. Now techniques have been developed using fucoidan as an ingredient in a film format [71]. In Cashman’s 2010 study, a *Fucus vesiculosis* fucoidan loaded film inhibited the number of adhesions in a rabbit model by up to 90%. The effective level of fucoidan loading in the film was in the range of 0.33% to 33% w/w and no toxicity was observed.

### 6.3. Liver Fibrosis

Liver fibrosis occurs after toxic or pathogen insult to the liver. The function of the liver is compromised as the fibrous tissue takes over from active liver cells and significant morbidity may ensue. Several recent publications indicate that fucoidan has protective effects against liver damage by inhibiting fibrosis and this can be achieved by oral dosing in the models tested.

In damaged or diseased livers, hepatic stellate cells proliferate into fibrous masses whereas functional hepatic cells expire. Hayashi’s 2008 research into carbon tetrachloride induced liver injury showed that fucoidan was a highly effective inhibitor of this type of fibrosis [72]. Saito used an alternative liver damage model (Concanavalin A), but used the same type of *Fucus vesiculosis* fucoidan [73] intravenously to inhibit the damage.

Nakazato’s research used a different model of liver damage, but illustrated a profound inhibition of damage by orally delivered high molecular weight fractions of *Cladosiphon okamuranus* fucoidan [21]. Both crude (28 kDa) and high molecular weight (41.4 kDa) fucoidan fractions were delivered at 2% w/v in drinking water over 12 weeks. The high molecular weight fraction was a powerful inhibitor of *N*-nitrosodiethylamine (DEN) induced liver fibrosis as measured by histology and staining. There was a strong down regulation of TGF beta and SDF1 expression in the liver as compared to the controls.

d-galactosamine-induced hepatopathy is used as a model for liver disease because it resembles acute viral hepatitis [74]. Kawano had previously identified therapeutic potential of edible whole seaweeds in the diet of experimental rats with this type of liver damage [75]. In his 2007 study he identified the components of the seaweeds that gave rise to the inhibition of liver disease. The elevated levels of aspartate and alanine aminotransferases were inhibited specifically by the fucoidan in the diet at levels equivalent to those in whole seaweed at 5% of the total diet [75,76]. Lastly, Hayakawa addressed the issue of hepatocarcinoma in *ex vivo* samples of chronic cirrhosis and hepatitis B and C from a clinical setting and found that *Fucus vesiculosis* fucoidan has a potentially therapeutic effect [77].

To conclude, fucoidan appears to have good potential as an orally delivered adjunct therapy for liver disease. See Table 4 for details.

### 6.4. Radiation and Fucoidan

Radiation causes a number of potential harms. Firstly, uptake of radioactive substances into the body perpetuates radiation damage, especially in children. Secondly, radiation damage itself causes cell death. This is particularly dangerous in that immune cells can be completely eliminated. The hemopoeitic stem cells that repopulate the immune system arise in the bone marrow and are called bone marrow stem cells. The protection of this progenitor population is very important in both medical and emergency settings.

Whole seaweed and seaweed extracts, particularly alginate, have been ingested as moderators of radiation damage [78]. The decontaminating effects of alginate syrups and additions to breads and milks have been known about for many years [79]. Alginate is used as a syrup useful in both reducing uptake of radioactive elements and increasing their excretion or “decorporation” [80]. Whilst iodine is a naturally occurring component and regular ingestion would assist in maintaining thyroid saturation, well timed and high levels of iodine in the form of sodium iodide are required to prevent uptake of radioactive isotopes of iodine.

Fucoidan, although a chelating agent, has not been recognized as a decorporation agent for radionuclides. It has, however, been demonstrated to provide radiation protection in both *in vitro* and mouse models [81,82]. Fucoidan was known to stimulate the maturation of dendritic cells, which are potent antigen-presenting cells in the immune system [83]. Byon measured the expression levels of cell surface markers in bone marrow stem cells after radiation. In this study the fucoidan was delivered intraperitoneally prior to radiation. As bone marrow is the main cellular source for the hematopoietic and immune systems, it contains large numbers of lymphocytes, granulocytes, and stromal cells as precursors and mature cells. Their results suggested that a specific population of cells—granulocytes— may selectively survive in response to fucoidan treatment following irradiation [82].

In irradiated mice the optimal intraperitoneal pre-treatment dosage of fucoidan to maximise survival was determined to be 100 mg/kg delivered into the peritoneal cavity [81]. Rhee also demonstrated the potential for protection from gamma irradiation in mice and *in vitro* [83]. *Laminaria japonica* derived fucoidan delivered intraperitoneally at 100 mg/kg was found to protect against changes in the counts of blood cells. Several parameters indicated better recovery after irradiation. The erythrocyte count in the irradiated controls ranged from 64% to 67% but the fucoidan-treated group increased gradually, ranging from 75% to 80%. The mean number of survival days and the 50-day survival rate in this group was 29 days and 30% at the highest fucoidan dose of 100 mg/kg as compared to 9 days and 0% for the control group.

### 6.5. Stem Cells and Fucoidan

Fucoidan is a known stem cell mobilizer when delivered intravenously. It has also shown activity then delivered orally [28]. When delivered intravenously, *Fucus vesiculosis* fucoidan is highly effective in mobilizing stem cells into the peripheral circulation in baboons and mice. The mechanism of mobilization appears not to relate solely to the inhibition of selectin binding but also to fucoidan’s ability to bind to SDF1 (CXCL12) [84,85]. Stem cell mobilization is used as a method of collecting sufficient immune repopulating cells from the peripheral circulation that are stored and re-engrafted after chemotherapy and radiotherapy in selected cancer patients.

SDF1 binds to the G protein coupled CXCR4 receptor, which has SDF1 as its unique ligand. CXCR4 is expressed by some hemopoietic stem cells, most leukocyte populations, endothelial cells, epithelial cells and some carcinoma cells. This SDF1/CXCR4 affiliation is thought to be involved not only in the regulation of stem cell release and homing, but also in some disease processes, including fibrogenesis [85].

The binding of fucoidan to SDF1 was found to block liver regeneration in an animal model [86]. This outcome contrasts with the marked radiation protection of hemopoietic stem cells by fucoidan described in Section 6.4 and the effective use of fucoidan in the inhibition of liver disease in a number of animal models as described in Section 6.3. In Mavier’s example, *Fucus vesiculosis* fucoidan was delivered intraperitoneally to rats at 25 mg/kg body weight. The liver was entirely damaged, both chemically and physically, and regeneration was observed. Under less dramatic circumstances, the liver regenerates from mature differentiated cells but when injured or after partial hepatectomy combined with inhibition of mature hepatocyte proliferation, regeneration is achieved from liver stem cells, which are known as “oval cells”. In the control damaged rats, oval cell accumulation could be seen, but in fucoidan treated rats, the oval cell numbers were severely depressed.

Oral delivery of *Undaria pinnatifida* fucoidan in a clinical observational study also resulted in an increase in the number of stem cells, of serum levels of SDF1 and the expression of CXCR4 on stem cells (CD34+) [28]. This observation correlates with the *in vitro* and animal studies showing that fucoidan mobilizes stem cells, although the degree of mobilization was not as large at the time point measured. The increase in CXCR4 expression could in some way reflect the binding of SDF1 by fucoidan.

Lastly, oral delivery of a seaweed extract in a clinical study consisting of a blend of *Fucus vesiculosis*, *Macrocystis pyrifera* and *Laminaria japonica* fucoidans increased phagocytosis by granulocytes and macrophages [33] in addition to NK and cytotoxic T cell numbers. Additionally the chronic inflammation marker interleukin 6 was lowered. This data has common features with an animal study carried out by Shimizu [38] and may point to the potential for radiation protection potential by orally delivered fucoidan.

### 6.6. Blood Homeostasis

It is beyond the scope of this review to consider the entire recent research on fucoidan fractions and blood homeostasis. In brief, fucoidan compounds have a retarding effect on coagulation in part due to interference with antithrombin III and heparin cofactor II [87]. The *in vivo* effects of intravenous delivery of most types of fucoidan are to extend global clotting time, an effect which is reversible [36]. Kwak recently investigated *Fucus vesiculosis* fucoidans inhibitory effect in a ferric chloride induced thrombosis model in mice [88]. Fucoidan showed a stronger antithrombotic effect than heparin. The anti-thrombin and anti-factor Xa activities of fucoidan *in vitro* were less than those of heparin, which implies that the antithrombotic activities are due to binding with heparin cofactor II rather than with antithrombin.

Pomin addresses the issues of strong anticoagulant activity of fucoidans by desulfation, producing fractions with desirable bioactivity unfettered by anticoagulant behaviors [89]. A linear sulfated fucoidan from sea urchin egg jelly required significantly longer chains than mammalian glycosaminoglycans to achieve the same anticoagulant activity. A slight decrease in the molecular size of the sulfated fucan dramatically reduced its effect on thrombin inactivation mediated by heparin cofactor II. A sulfated fucoidan with 45 tetrasaccharide repeating units was able to bind to heparin cofactor II but was unable to link efficiently the plasma inhibitor and thrombin, which required fucoidan with 100 or more tetrasaccharide repeating units.

A recent clinical study examined the effects of orally ingested fucoidan on clotting times. 3 g of *Undaria pinnatifida* fucoidan were ingested daily for 12 days by healthy subjects [29]. A significant prolongation of global clotting time was noted but this was within normal clinical parameters. Toxicological studies on *Undaria pinnatifida* fucoidan in rats showed no effect on clotting times, even at very high doses [25]. A toxicology study on *Laminaria japonica* fucoidan did show some pertubations in clotting at 900 mg/kg [26], far above the dose used in the clinical study.

More recent research into activity illustrates the somewhat counter-intuitive “procoagulant” effect of fucoidan in hemophilia model [31]. In hemophilia A dogs, orally delivered *Laminaria japonica* fucoidan fraction improved clotting times, perhaps by creating a bypass in the normally blocked clotting pathway.

### 6.7. Neuronal Protection

Increasing interest in reducing or reversing brain ageing and disease has seen many agents assessed for their effects on neurons and brain function. Alzheimer’s is a brain disease that causes significant and highly distressing loss of function. It is characterized by the accumulation of beta amyloid plaques in the brain tissue. It has been hypothesized that Alzheimer’s is associated with Herpes infections in the central nervous system [90]. The relatively large size of fucoidan precludes penetration of the blood brain barrier but as noted below, systemic administration was effective in maintaining neuronal function in a mouse model.

Fucoidan shows promise in protecting brain function in a number of ways. It has been comprehensively assessed by Jhamandas [91] for effects on current in whole rat brain neurons and also on the neurotoxic effects caused by beta amyloid in primary culture neuronal cells. *Fucus vesiculosis* fucoidan at one micromolar concentration could inhibit currents by 15%. It was also able to reverse the current blocking effects of beta amyloid. Fucoidan did not inhibit the aggregation of beta amyloid, but did protect primary cultures of rat basal forebrain cholinergic neurons, against amyloid induced cell death.

Cui’s recent research centered on a lipopolysaccharide (LPS) activated microglial cells [92]. Microglia are usually resting cells in the brain, but if activated, for example by LPS, they change shape, become active and secrete inflammatory mediators and nitric oxide (NO). If too much activation takes place, surrounding neuronal cells die, instigating more inflammation and beginning a cycle of neurodegeneration. 62.5 mcg/mL fucoidan was able to prevent shape change in the LPS exposed microglial cultures, and 125 mcg/mL inhibited NO production. In 2004 Li demonstrated the characteristic fragmented DNA (fDNA) observed in neuronal nuclei in Alzheimer brain was taken up by adjacent activated microglia which then became activated [93]. Blocking the scavenger receptors on the microglial cells with *Fucus vesiculosis* fucoidan at 40 ng/mL suppressed this uptake.

Pei also demonstrated the scavenger receptors on microglial cells could be successfully blocked by fucoidan [94]. Huang [95] used time lapse recording to investigate how microglia are involved in the growth of amyloid plaques. *Fucus vesiculosis* fucoidan at 10 micromolar concentration was able to inhibit the amyloid induced microglical clustering effect that gave rise to plaques. Do presented a study on tumor necrosis factor alpha (TNF-alpha) and interferon gamma (IFN-gamma) induced NO production in glioma cells [96]. Fucoidan inhibited NO production in this model system via a number of signaling pathways.

Luo, from the same research team as Cui, investigated the effects of the same *Laminaria japonica* fucoidan fraction in a model of Parkinson’s disease in mice [97]. Mice were given fucoidan at a dose rate of 12.5 mg/kg or 25 mg/kg, intraperitoneally daily for 18 days. The Parkinson’s inducing agent was given on the 11th day an hour after injection of fucoidan. There was a clear dose dependent protection by fucoidan at 25 mg/kg body weight, including inhibition of lipid peroxidation, enzyme functions and glutathione levels. See Table 5.

### 6.8. Viruses: Influenza, Herpes and Dengue

Inhibitory effects of fucoidan on coated viruses are well known and were well reviewed a decade ago by Schaeffer [100]. Fucoidans have no direct pathogen killing activity but rather, they inhibit infection via receptor entry blocking and interference with replicative processes. Other sulfated polysaccharides in addition to fucoidans have been known to possess inhibitory activity against coated viruses such as Herpes and HIV [100–102]. In the case of viruses, receptor blocking activity prevents viral cell entry and there may also be inhibition of viral replication and syncytia (giant cell) formation. Drawbacks to using fucoidan as an orally delivered agent remain those of bioavailability. However, pathogens targetable from the lumen of the gut, such as viruses harbored in gut immune structures, may be particularly well targeted by oral fucoidan. As the following examples illustrate, oral dosing does have protective effects against viral infection.

Influenza epidemics have become topical in recent years with outbreaks of Influenza A causing significant global concern. Existing therapies can become useless as resistance develops. Several research groups have investigated inhibitory effect of fucoidan fractions on clinical strains of influenza [103] and para-influenza [104] *in vitro. Undaria pinnatifida* has a marked inhibitory effect on the recent H1N1 influenza A virus with half maximal effective inhibitory concentrations at less than 1 mcg/mL [44]. Notably, these effects can also be seen *in vivo* with orally delivered fucoidan. Hayashi reported mouse model data in which 5 mg per day orally delivered *Undaria pinnatifida* fucoidan strongly inhibited influenza A infection [44]. It had a marked synergistic effect with oseltamivir, a neuraminidase inhibitor effective in treating influenza. Even as a sole agent, fucoidan was able to significantly reduce viral titres in the lung of infected mice. At the time of writing, there are no clinical studies on Influenza using fucoidan reported in the available literature.

Herpes infections can be debilitating and recurrent. There is a need for a both treatment and prevention strategies for both Herpes 1 and 2. In a small clinical case study series, Herpes reactivation was inhibited by ingesting a fucoidan rich *Undaria pinnatifida* preparation [105]. *Undaria pinnatifida* fucoidan was shown to be a highly effective inhibitor of clinical strains of Herpes entry to cells in culture [102,106] at microgram concentrations. Interestingly, stains resistant to the common drug therapy, acyclovir, were equally well inhibited. Hayashi established that the inhibitory effect of fucoidan was carried over to *in vivo* setting in a mouse model [101]. In his research, orally delivered *Undaria pinnatifida* fucoidan exerted protective effects via direct inhibition of viral replication and stimulation of both innate and adaptive immune defense functions. Oral administration of the fucoidan at 5 mg/day every 8 h (three times per day) from 1 week before virus inoculation to 1 week post-inoculation (pretreatment), or for 1 week after virus inoculation protected mice from infection with HSV-1 and enhanced cytotoxic T cell activity. NK cell activity was improved in HSV-1-infected immunosuppressed mice. The production of neutralizing antibodies in the mice inoculated with HSV-1 was significantly promoted by the oral administration of the fucoidan for 3 weeks.

Dengue virus is a debilitating viral infection spread by mosquitoes. Sometimes known as ‘bone crack fever’ for its painful joint effects, it has four different subtypes. Infection by one does not confer protection from infection by another type. There are no treatments for dengue other than pain relief and good nursing. Fucoidan from *Cladosiphon* has been shown to inhibit type 2 dengue *in vitro* [107]. Given the effectiveness of oral fucoidan in inhibiting Herpes, there may be potential in further research into the inhibitory effects of fucoidan fractions on Dengue fever. At the time of writing, there are no clinical studies on dengue using fucoidan reported in the literature.

### 6.9. Viruses: HTLV1

In a clinical “first” this year, Araya presented data on orally delivered fucoidan to treat Japanese subjects infected with Human T lymphotropic virus type-1 (HTLV-1) [34]. This human retrovirus causes HTLV-1associated myelopathy/tropical spastic paraparesis (HAM/TSP) and is also responsible for adult T cell leukaemia in a small number of infected subjects. A higher viral load in individuals with HTLV-1 infection increases their risk of developing and the clinical progression of HAM/TSP and unpleasant and increasingly disabling condition. *In vitro* studies by Japanese and Brazilian researchers have indicated that fucoidan from *Cladosiphon okamuranus*, and fucoidan containing water extracts from *Sargassum vulgare* and *Laminaria abysslis* were effective at inhibiting HTLV-1 viral infection in cell culture [108,109]. Although the extracts were not selectively toxic to infected cells, syncytia formation and thus cell to cell transfer of the virus was inhibited. In Araya’s 2011 study, the *in vitro* inhibitory effect of fucoidan on cell-to-cell HTLV-1 infection was confirmed by using luciferase reporter cell assays.

In Araya’s open-label trial, 13 patients with HAM/TSP were treated with 6 g of a relatively pure (85%) *Cladosiphon okamuranus* derived fucoidan daily for 6–13 months. HTLV-1 proviral DNA load was measured and found to decrease by 42.4% on average over the trial period. Interestingly, there was variation in the trial subjects with some achieving much greater reductions. Araya also measured immune cell function, as fucoidans are known to modulate immune function in other animal [38–40,53] and clinical studies [33,53]. Frequencies of HTLV-1-specific CD8(+) T-cells, natural killer cells, invariant natural killer T-cells and dendritic cells in the peripheral blood were analyzed but did not show significant changes. This contrasts with research in which fucoidan intake did alter immune parameters [33,40]. In this clinical study, there were mild side effects. During the treatment four patients with HAM/TSP developed diarrhea, which improved immediately after stopping fucoidan administration.

### 6.10. Leishmaniasis

Leishmaniasis is a protozoan parasite spread by the bites of sandflies. As many as 12 million people are believed to be currently infected, with about 1–2 million estimated new cases occurring every year [110]. There are many different types of leishmaniasis, most being spread from animal to humans, but some species are also spread between humans. Leishmaniasis most commonly manifests as a skin disease causing ulcers but may also spread to organs, a condition known as “visceral leishmaniasis” and can be fatal. There are no vaccines for leishmaniasis, and one older treatment, sodium antimony gluconate, is no longer effective in India.

In 2011, Kar published research in which fucoidan was administered in a rat model to treat Leishmaniasis [111]. Whilst the authors are cautious about the extrapolation of their work to humans and to other types of leishmaniasis, the potential for an adjunct or even a sole therapy is strong. Their data showed inhibitory effects on amastigote (part of the life cycle of leishmaniasis) multiplication within macrophages. There was complete elimination of liver and spleen parasite burden at 200 mg/kg/day given orally, 3 times weekly, in a 6-week mouse model of both antimony-susceptible and -resistant strains. They noted switching of immune defense from a Th2 to Th1 mode and increased splenocyte superoxide and NO production. IL-10 and TGF beta, both Th1 suppressive cytokines, were found to be profoundly down-regulated in infected fucoidan-treated mice. TGF beta, was found to be significantly down-regulated in fucoidan-treated mice. Most importantly, fucoidan was thought to be curative when administered orally 15 days post-infection.

At the time of writing there are no published clinical studies involving fucoidan and leishmaniasis infections.

### 6.11. Malaria

According to the World Health Organization, in 2008, there were 247 million cases of malaria and nearly one million deaths. Malaria is caused by four different types of *Plasmodium* parasites. The parasites are spread to people through the bites of infected *Anopheles* mosquitoes, called “malaria vectors”. If resistance to the currently effective artemisinin drugs develops and spreads to other large geographical areas, as has happened before with chloroquine and sulfacoxine-pyrimethamine the public health consequences could be grim, as no alternative antimalarial medicines will be available in the near future.

The malaria parasites (*Plasmodium* merozoites) enter the blood stream and must then penetrate a red blood cell (erythrocyte) in order to multiply. The cell bursts and the cycle begins again. Malaria leads to severe anemia and cycles of fever as the parasite takes hold. Fucoidan is one agent that shows some promise as an inhibitory agent for *Plasmodium* infection *in vitro* and in mouse models [112]. *Undaria pinnatifida* fucoidan significantly inhibited the invasion of erythrocytes by *Plasmodium falciparum* merozoites. Four-day suppressive testing in *Plasmodium berghei*-infected mice (a model system) with fucoidan resulted in a 37% suppressive effect *versus* the control group and a significant delay in the deaths from anemia.

At the time of writing, there are no clinical studies on malaria using fucoidan reported in the available literature.

### 6.12. Prions

Transmissible spongiform encephalopathies or “prion” diseases are a group of fatal neurodegenerative disorders. They include the human diseases “Kuru” and Creutzfeldt-Jakob disease (CJD), the sheep disease scrapie, and bovine spongiform encephalopathy which affects cattle. ‘Kuru’ occurs among people from New Guinea who practiced a form of cannibalism in which they ate the brains of dead people as part of a funeral ritual. This practice stopped in 1960, but cases of Kuru occurred for decades because of the long incubation period [113,114]. These diseases are characterized by accumulation of an abnormally folded prion protein in the central nervous system and the lympho-reticular system. Recently, variant CJD became a major health concern in Europe after infected meat entered the food chain.

Prions remain a therapeutic challenge with no approved preventative or curative drug therapy available. Doh-Ura and colleagues examined the effects of fucoidan ingestion on prion infection in a mouse model and illustrated that prior ingestion of fucoidan could inhibit the development of disease [115]. Fucoidan orally administered to the mice after the enteral inoculation delayed the disease onset for about half the time of the control incubation. However fucoidan administered before enteral inoculation did not affect the incubation time. As fucoidans are large molecules, the blood brain barrier is unlikely to allow passage into the central nervous system. It is perhaps more likely that fucoidan exerted effects via the gut immune system. To date there are no reported clinical studies with fucoidan to assess effects on prion infections.

### 6.13. Helicobacter and Stomach Ulcers

A recent encouraging clinical study was carried out in Indonesia to determine the effectiveness of fucoidan therapy for *Helicobacter pylori* affected patients [116]. This study was supported by considerable *in vitro* and *in vivo* evidence to suggest that fucoidan has inhibitory effects on *Helicobacter* infections, largely by inhibiting adhesion to mucosal surfaces [117]. Juffrie’s study included patients with at least eight years of abdominal pain or dyspepsia. After endoscope confirmation of ulceration and *Helicobacter* infection, subjects took placebo or 100 mg *Cladosiphon okamuranus* fucoidan daily for three weeks before undergoing another endoscopy which included a biopsy. There was relief from symptoms such as pain and vomiting in the fucoidan treated patients from 5 days into the study. At biopsy, the majority of the patients with severe ulcers who received fucoidan at 100 mg per day for three weeks changed to the healing stage or scarring stage whereas only 37.5% patients who received placebo changed to the healing stage.

In animal models, aspirin induced ulceration (a model for gastric ulcers) could be inhibited by orally delivered *Fucus vesiculosis* fucoidan at dose rates of 20 mg/kg body weight/day for two weeks [118]. Aspirin treatment generated the expected rises aspartate and alanine transaminases (AST and ALT) and the serum cytokines IL6, IL10 and IFN gamma. Oral fucoidan showed considerable (*p* < 0.05) protection against ulceration by inhibiting the acute alterations of AST, ALT, cytokines and stomach glycogen. However, aggravated serum INF-gamma was observed in the fucoidan-pre-treated group [118].

### 6.14. Renal Disease and Hyperoxaluria

Fucoidan may act in several ways to protect against kidney damage. As for other organs in the post ischemic stage, reperfusion injury may be protected by *Fucus vesiculosis* fucoidan administration at 10 mg/kg intravenously on the basis of its ability to inhibit P selectin [119]. Zhang demonstrated the effects of up to 200 mg/kg body weight orally administered *Laminaria japonica* fucoidan in the prevention of chronic renal failure or Heymann nephritis (an autoimmune condition) in a rat model [120,121].

Elevated levels of oxalate in serum are commonly associated with the painful joint swellings known as gout. Elevated oxalate levels are also responsible for kidney stones. In 2007, Veena assessed *Fucus vesiculosis* fucoidan in Wistar rats in which hyperoxaluria and calcium oxalate deposition in the kidneys was induced by ethylene glycol [122]. Daily doses of 5mg/kg body weight were administered subcutaneously over 28 days, after which tissues were harvested for assessment. Fucoidan successfully normalized the disturbances associated with hyperoxaluria. Josephine, from the same research group, published additional research in which the effects of fucoidan on Cyclosporine A (CsA)-induced nephrotoxicity were assessed [123]. A dose of 5 mg/kg body weight fucoidan isolated from *Sargassum wightii* or *Fucus vesiculosis* was administered subcutaneously over 21 days. Both fucoidans protected mitochondrial function and other biological measures to the same degree. The reasoning behind the model was the therapeutic use of cyclosporine. Cyclosporine is a useful immunosuppressive during transplants yet its nephrotoxicity curtails the degree of its use.

### 6.15. Snake Envenomation

Snake bite is a seriously neglected issue affecting Central and South America, parts of Asia and Africa [124–126]. Existing therapy for snake bites consists of anti-venoms. These can be costly, have a limited shelf life and also require careful storage. They are not always available in areas of greatest need.

Snake venoms are a complex mixture that may cause swelling, bleeding, blistering, and ultimately skin and muscle necrosis. In bites from commonly occurring South American snakes these effects are caused by two major components of the venoms; zinc dependent metalloproteinases and myotoxic phospholipases (PLA2). The metalloproteases degrade extracellular matrix components, such as the collagens that make up microvessel structures. The myotoxic phospholipases break down the plasma membrane of skeletal muscle cells. A large inflammatory reaction ensues with infiltration of leukocytes. Swelling follows, which can cause restricted blood flow, ischemia and further tissue damage.

Fucoidan from *Ascophyllum nodosum* was been investigated by Costa Rican research teams and was found to be an effective inhibitor of PLA2 variants present in the venoms of crotalid snakes [124–127]. Crotalid snake venom damages the lymphatic system via the enzyme PLA2. Mora demonstrated that the potent PLA2 inhibitory effects of fucoidan could completely eliminate the myotoxic effects of the venom if preincubated with it prior to injection in a mouse model, but it did not change the hemorrhagic effects of the venom [125]. Angulo demonstrated that fucoidan forms complexes with the snake venom PLA2 and inactivates them [127].

In Rucavado’s most recent study [124], the effect of fucoidan combined with batimastat (a peptidomimetic hydroxamate metalloproteinase inhibitor known to inhibit crotalid venom activity) was investigated in a mouse model. Fucoidan reduced the presence of intracellular proteins in exudates, whereas batimastat reduced the amount of relevant extracellular matrix proteins. The combination of these inhibitors resulted in the elimination of the most relevant pathological effects of this venom.

Curiously, lowering the molecular weight of the fucoidan to theoretically enhance bioavailability was not effective in inhibiting snake venom [42]. A study evaluated the ability of two fractions of *Ascophyllum nodosum* fucoidan to prevent muscle necrosis when rapidly administered after injection of a purified myotoxin or crude venom in a mouse model. The two preparations were standardized to the same neutralizing potency. Local administration into the muscle of either fraction after myotoxin injection prevented nearly 50% of muscle necrosis. However, when tested against crude venom (which contains several myotoxin isoforms), muscle injection of the higher molecular weight fraction inhibited myonecrosis by nearly 50% as compared to the untreated group. In contrast to expectations, the use of smaller fucoidan fragments reduced rather than increased the therapeutic response. This may relate to a particular structural feature in the high molecular weight fraction that is absent in the lower molecular weight fraction.

In 2003, Vasanthi described the successful inhibition of cobra snake venom in a mouse model by intraperitoneal administration of an extract from *Padina boergensii* [128]. However, the extract was prepared with methanol, and it is unclear whether fucoidan was a major component of the extract as fucoidan has limited solubility in alcohols.

The relative stability of fucoidan fractions compared to conventionally produced antivenoms means that no cold storage would be required and a long shelf life could be anticipated. The development of fucoidan for this application could potentially limit the complications and distress caused by snakebite in a cost effective and realistic manner.

## 7. Conclusions

This review covers some of the more recent research into the bioactivity and discusses potential therapies from fucoidan. The research field has increased in the last decade and has started to produce clinical studies. This review has spanned potential applications as diverse as prion infection and osteoarthritis. The development of fucoidan fractions requires attention to the source and the required characteristics of the fraction, in addition to consideration of the route of delivery. Oral delivery appears promising, with research indicating therapeutic potential in different areas. Increasing bioavailability is likely to be important for orally delivered fucoidan. Overall, the availability and safety of commercially available fucoidan preparations will lead to interesting adjunct and sole therapies for some neglected disease states in addition to providing new approaches to inflammation and fibrosis.

## Figures and Tables

**Table 1:** Antibodies to fucoidan.

Specificity of antibody	Seaweed source	Trial	Route of administration	Use	Reference
Heparin and fucoidan	*Undaria pinnatifida*	Human	Oral	Estimation of serum uptake	Irhimeh 2005 [19]
Fucoidan	*Cladosiphon okamuranus*	Human	Oral	Estimation of serum, urine concentration	Tokita 2010 [20]
Fucoidan	*Cladosiphon okamuranus*	Rat	Oral	Staining in liver	Nakazato 2009 [21]
Fucoidan	*Laminaria japonica* and *Kjellmania gyrate*	Botanical stain	n/a	Staining	Mizuno 2009 [23]

**Table 2:** Toxicology of fucoidan.

Fucoidan source	Characteristics	Studies	Dose	Species	Results	Ref.
*Undaria pinnatifida*	53% total sugar 7.4% sulfate 27% Uronic acid 54% fucose 35% galactose	Acute *in vivo* Ames test Bone marrow micronucleus	1000 mg/kg body weight per day for 28 days	Sprague Dawley rats	Not toxic to 1000 mg/kg bw; Increase in ALT at 200 mg/kg	Chung 2010 [25]
*Laminaria japonica*	Fucoidan Fraction MW average “10–300 kD”	Oral dosing in experimental model; Toxicity by clinical observation	Escalation doses up to 20 mg/kg	Dogs with hemophilia A; Rats	No clinical toxicity	Prasad 2008 [31]
*Laminaria japonica*	Fluorescent labeled fucoidan	Subcutaneous	5 mg/kg	Rat	Half life 83 min	Prasad 2008 [31]
*Laminaria japonica*	MW average 189 kDa Fucose 28% sulfate 29% total sugasr 48%	Acute and sub-chronic toxicity	300, 900 and 2500 mg/kg bw to 6 months	Wistar Rats	Not toxic to 300 mg/kg bw Prolonged clotting at 900 and 1200 mg/kg bw	Li 2005 [26]
*Undaria pinnatifida*	64.4 ± 6.0% fucose, 31.9 ± 4.7% galactose, 3.6 ± 1.3% mannose, and 31.7 ± 2.2% sulfate	Genotoxicity Bacterial mutation Bone marrow Micronucleus formation	Up to 2000 mg/kg/bw orally	Sprague Dawley rats	Fucoidan presents no significant genotoxic concern.	Kim 2010 [32]
*Undaria pinnatifida*	64.4 ± 6.0% fucose, 31.9 ± 4.7% galactose, 3.6 ± 1.3% mannose, and 31.7 ± 2.2% sulfate	Toxicty measures—body weight, ophthalmoscopy urinalysis, hematology, and histopathology; Clotting paramaters; Prothrombin time or activated partial thromboplastin time	1350 mg/kg bw/day for 4 weeks orally	Sprague Dawley rats	No changes to No change to prothrombin time or activated partial thromboplastin time	Kim 2009 [30]
*Undaria pinnatifida*	75% fucoidan	Full blood count, clinical biochemistry	3 g per day for 12 days	Human	No toxicity noted	Irhimeh 2005, 2007, 2009 [19,28,29]
*Fucus vesiculosis* *Macrocystis pyrifera* *Laminaria japonica*	75% total fucoidan	Full blood count, clinical biochemistry	0.1 and 1 g for 84 days	Human	No toxicity	Myers 2010 [27]
*Fucus vesiculosis* *Macrocystis pyrifera* *Laminaria japonica*	75% total fucoidan	Full blood count, clinical biochemistry	0.1 and 1 g for 28 days	Human	No toxicity	Myers 2011 [33]
*Cladisiphon* sp.	85% fucoidan	Patients with HTLV1 were treated clinically	6 g per day up to 13 months	Human	Four patients diarrhea; No other toxicity noted; Reduction in viral load	Araya 2011 [34]

**Table 3:** Arthritis and fucoidan.

Fucoidan	Model	Adminstration	Observation	Reference
*Fucus vesiculosis* *Macrocystis pyrifera* *Laminaria japonica*	Human clinical	Oral daily 100 mg or 1000 mg for three months	No toxicity Up to 52% reduction in symptoms	Myers 2010 [27]
*Fucus fucoidan* (Sigma)	Mouse with surgical destabilization of the medial meniscus	20 mg/kg intraperitoneal injection	Fucoidan suppressed cellular infiltration of joint and suppress post-operative (3 days), but not late (16 weeks) pain	Mcnamee 2010 [70]
*Fucus fucoidan* (Sigma)	Wistar rats with carrageenan induced local inflammation in the footpad	20 mg/kg intravenously	Fucoidan inhibits mechanical hypernociception and neutrophil migration	Cunha 2008 [69]
*Undaria pinnatifida fucoidan*	Collagen induced arthritis in mice	100 kDa, 3.5 kDa and 1 kDa fractions orally administered daily 300 mg/kg to 49 days	1 kDa fraction effectively inhibited whereas 100 kDa exacerbated disease	Park 2010 [41]

**Table 4:** Liver disease and fucoidan.

Fucoidan	Model	Treatment	Result	Ref.
*Fucus vesiculosis* fucoidan (Sigma)	Concanavalin induced liver injury in mice	1–30 mg/kg intravenously 30 min before concanavalin A	Significantly inhibited raised levels of TNF-alpha and IFN-gamma. Increased endogenous IL-10 production	Saito 2006 [73]
*Fucus vesiculosis* fucoidan (Sigma)	CCl4 induced liver fibrosis in mice	fucoidan (50 mg/kg body weight) administered intraperitoneally for 8 weeks	Protection of normal hepatocytes. Inhibition of hepatic stellate cell proliferation	Hayashi 2008 [72]
*Cladosiphon okamuranus fractions* Crude (28 kDa) and HMW (41.4 kDa)	*N*-nitrosodiethylamine (DEN) induced liver fibrosis model in Sprague dawley rats	2% fucoidan in drinking water for 12 weeks	Protection from damage Increased metallothionein Down regulation of TGFbeta 1 and SDF1	Nakazato 2010 [21]
*Fucus vesiculosis* fucoidan (Sigma)	*Ex vivo* human hepatoma (hepatitis B and C) and Cirrhosis	Inhibition of biotidinase in tissue samples was assessed	Fucoidan decreased the disease elevated activity of biotinidase	Hayakawa 2009 [77]
*Fucus vesiculosis* fucoidan (Sigma)	Rats with d-galactosamine-induced hepatopathy	Dietary inclusion of ~1.2% fucoidan for 8 days	Fucoidan was protective against d-galactosamineinduced hepatopathy in rat	Kawano 2007 [75,76]

**Table 5:** Brain cells and fucoidan.

Fucoidan type	Model	Effects	Reference
*Fucus vesiculosis* fucoidan (Sigma)	Rat neuron *in vitro*	Inhibits neurotoxic effects of amyloid	Jhamandas 2005 [91]
*Laminaria japonica* fuucoidan average MW 7000	Lipopolysaccharide activated microglial cells *in vitro*	Inhibits NO production in LPS activated microglial cells	Cui 2010 [92]
Sea cucumber fucoidan	Neuronal stem cells *in vitro*	Protection from radiation	Zhang 2010 [98]
*Laminaria japonica* fuucoidan average MW 7000	MPTP-induced Parkinsonism in mice Fucoidan delivered IP	Protects against Parkinsons disease model of neuronal damage	Luo 2009 [97]
*Fucus vesiculosis* fucoidan (Sigma)	Microglial cells *in vitro*	Fucoidan completely blocks microglial uptake of fDNA at only 40 ng/mL	Li 2004 [93]
*Fucus vesiculosis* fucoidan (Sigma)	*In vitro* microglial clustering assay	Fucoidan inhibits beta amyloid induced microglial clustering at 10 μM	Huang 2009 [95]
*Fucus vesiculosis* fucoidan (Sigma)	Microglial cells *in vitro*	Inhibits LPS uptake into microglia by scavenger receptors	Pei 2007 [94]
*Fucus vesiculosis* fucoidan (Sigma)	Glioma cells *in vitro*	Fucoidan inhibits TNF-alpha- and IFN-gamma-stimulated NO production via p38 MAPK, AP-1, JAK/STAT and IRF-1	Do 2009 [96]
*Fucus vesiculosis* fucoidan (Sigma)	NO production in neuronal blastoma cells *in vitro*	Fucoidan has protective effect via inducible nitric oxide synthase (iNOS)	Lee 2007 [99]
